# Design of a Yellow-Emitting Phosphor with Enhanced Red Emission via Valence State-control for Warm White LEDs Application

**DOI:** 10.1038/srep31199

**Published:** 2016-08-11

**Authors:** Jian Chen, Yangai Liu, Lefu Mei, Peng Peng, Qijin Cheng, Haikun Liu

**Affiliations:** 1 Beijing Key Laboratory of Materials Utilization of Nonmetallic Minerals and Solid Wastes, National Laboratory of Mineral Materials, School of Materials Science and Technology, China University of Geosciences, Beijing 100083, china; 2 Department of Materials Processing and Control Engineering, School of Mechanical Engineering and Automation, Beihang University, Beijing, 100191, China; 3 School of Energy Research, Xiamen University, Xiamen 361005, Fujian, China

## Abstract

The phosphor-converted warm W-LED have being rapidly developed due to the stringent requirements of general illumination. Here, we utilized a strategy to synergistically enhance the red region and emission intensity of novel Eu-activated yellow-emitting LaSiO_2_N phosphors. This was realized by predicting optimum crystal structure, and governing the concentration of doping ions as well as preparation temperature. By using these straight-forward methods, we were able to vary the valence to enhance the red region and improve the quantum efficiency of LaSiO_2_N phosphor. The warm W-LED lamp fabricated with this red region enhanced LaSiO_2_N:Eu phosphor exhibited high CRI (Ra = 86), suitable CCT (5783 K) and CIE chromaticity (0.33, 0.36), indicating this synergistically enhanced strategy could be used for design of yellow-emitting phosphor materials to obtain warm W-LEDs.

Due to the deficiency of red color, the cool and bluish-white light LEDs (typically a blue-light LED chip coupled with yellow-emitting YAG: Ce^3+^ phosphor) with high correlated color temperature (CCT) and low color rendering index (CRI) are gradually replaced by blue, green and red (RGB)-emitting phosphors with a UV/NUV chip^1^,^2^. However, high cost and poor luminous efficiency of RGB-emitting phosphor became the main obstacles of their popularization because of self-adsorption occurring among these phosphor particles^3^. Currently, the white LEDs (W-LED) packaged by NUV chips (365–420 nm) with mixed blue and yellow-red emitting phosphors have attracted much attention because of their high CRI, tunable CCT and CIE chromaticity coordinates^4^,^5^,^6^,^7^,^8^,^9^,^10^,^11^. Therefore, designing and developing tunable yellow-red emitting phosphors which can be effectively excited with NUV light are in great demand for W-LED industry.

Currently, La–Si–O–N system doped with Ce^3+^ ions has been widely reported as blue phosphors for the application in solid-state lighting, fluorescent lamps or plasma display panels (PDPs)^12^,^13^. The emission properties of this La–Si–O–N system doped with Ce^3+^ or Eu^2+^ strongly depend on Si/La and N/O ratios, because the 5*d* electrons of Ce^3+^ and Eu^2+^ ions are unprotected and sensitive to the change of the strength of crystal field and covalency^14^,^15^. According to the crystallographic examination for an equal amount of Ce^3+^ substitution, the degree of covalency increased in a sequence of La_5_Si_3_O_12_N < La_4_Si_2_O_7_N_2_< La_2_Si_6_O_3_N_8_ < LaSiO_2_N^16^. As supported by Dorenbos^17^, the emission position depended on nephelauxetic effect, crystal-field splitting (CFS), and Stokes shift. Herein, Eu^2+^ ions in LaSiO_2_N should have stronger nephelauxetic effect due to its high covalency, compared with other La–Si–O–N system compounds. This effect would shift the centroid of the *5d* band of Eu^2+^ ions to lower energy and result in the redshift of emission peak. Meanwhile, the higher formal charge of N^3−^ compared with O^2−^ makes the CFS become larger, and the rigid lattice would lead to a smaller Stokes shift^18^. Thus, the LaSiO_2_N doped with lanthanide, especially Eu^2+^, may emit long-wave bands. But until now, the Eu^2+^ photoluminescence in La–Si–O–N system has been rarely reported due to the charge mismatch of Eu^2+^ and La^3+^. However, in our recent experiment, we observed the Eu^2+^ photoluminescence in LaSiO_2_N, and this novel LaSiO_2_N: Eu phosphor, as expected, exhibited broad emitting in yellow region. Further, we designed a strategy to cooperatively enhance the red region and emission intensity of this phosphor via altering the concentration of doping ions and preparation temperature for high CRI warm W-LED application. The red region enhancements were caused by varied valence which would affect the energy level of 5*d* band of Eu^2+^ and charge-transfer state of Eu^3+^, resulting in redshift and the increase of absorption efficiency as well as energy transfer from Eu^2+^ to Eu^3+^. Fabricated by using a blend of LaSiO_2_N: Eu and commercial blue phosphors (BAM: Eu^2+^) with a 385 nm NUV LED chip, a warm white light with high CRI as well as suitable CCT and CIE chromaticity can be obtained.

## Results and Discussion

**Figure 1:**
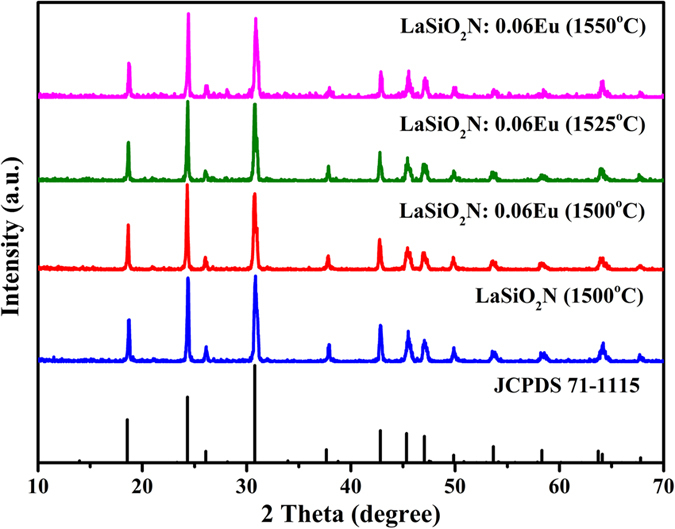
XRD patterns of as-synthesized LaSiO_2_N:Eu phosphors, LaSiO_2_N host and the standard pattern (JCPDS 71–1115) of LaSiO_2_N.

The phase purity of the as-prepared LaSiO_2_N:Eu phosphors and LaSiO_2_N host were substantiated by powder X-ray diffraction. As the Fig. 1 illustrated, all the diffraction peaks matched well with the standard pattern (JCPDS 71–1115) of LaSiO_2_N, demonstrating that the doping of Eu ions and the increased preparation temperature did not significantly influence its crystal structure.

**Figure 2:**
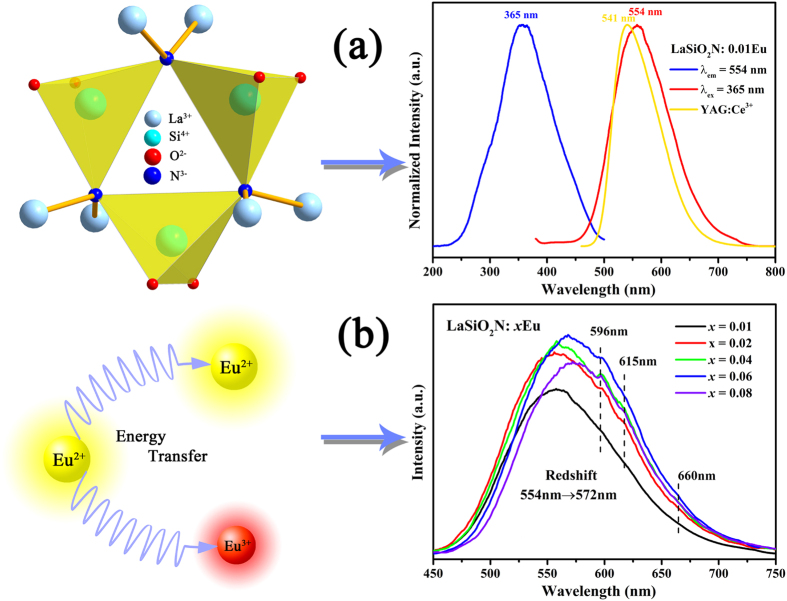
(**a**) Crystal structure, PLE (λ_em_ = 554 nm) and PL (λ_ex_ = 365 nm) spectra of the LaSiO_2_N:0.01Eu phosphor (prepared at 1500 °C); (**b**) Eu concentration-dependent PL (λ_ex_ = 365 nm) spectra of LaSiO_2_N:*x*Eu (*x* = 0.01, 0.02, 0.04, 0.06, 0.08) phosphors (prepared at 1500 °C), and the corresponding schematic illustration.

Figure 2a presents the crystal structure, photoluminescence excitation (PLE) and photoluminescence (PL) spectra of the LaSiO_2_N:0.01Eu phosphor prepared at 1500 °C. The LaSiO_2_N crystallizes as a hexagonal structure whose space group is P-6c2, and lattice constants are a = 7.31 Å, c = 9.550 Å, and V = 441.95 Å^3^. LaSiO_2_N has the α-wollastonite structure with N atoms present in the three-membered (Si_3_O_6_N_3_) rings, occupying bridging sites between pairs of Si-centered tetrahedral and linking to two La atoms^19^. Hence, the N environment is more ionic, and the lattice is more rigid than that of Si_3_N_4_ or Si_2_N_2_O. Based on the crystal structure, the LaSiO_2_N:Eu phosphor could be predicted to emit broad and long-wave band, and the following studies would verify this inference.

As shown in Fig. 2a, The PLE spectrum of LaSiO_2_N:0.01Eu monitored by 554 nm was composed of a broad excitation peaked at 365 nm ranging from 250–500 nm, which can be attributed to the 4f ^7^(^8^S7/2)–4f ^6^5d^1^ transition of Eu^2+^ ions^20^, and matched well with the emission of commercial N-UV chip (365–420 nm). The reduction of Eu^3+^ to Eu^2+^ in the trivalent La site can be explained with the charge compensating defect in the first anion (O^2−^ ion) coordination shell^17^. The PL spectrum of LaSiO_2_N:0.01Eu under 365 nm light excitation exhibited a broad yellow band from 450 to 750 nm peaked at 554 nm with a full width at half-maximum (FWHM) of 115.87 nm which was larger than that of YAG phosphor (91.65 nm), indicating that the synthesized phosphor here is a suitable yellow-emitting phosphor candidate for W-LED application.

**Figure 3:**
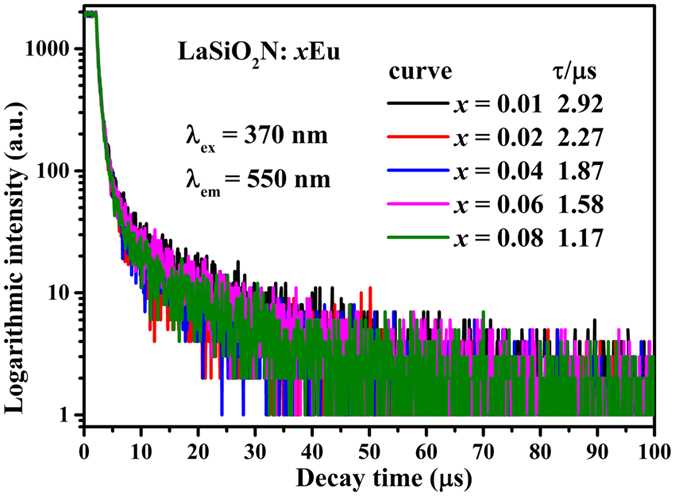
The decay curves of LaSiO_2_N:*x*Eu (*x* = 0.01, 0.02, 0.04, 0.06, 0.08).

Figure 2b depicts the Eu concentration dependent PL spectra of LaSiO_2_N:*x*Eu (*x* = 0.01, 0.02, 0.04, 0.06, 0.08) phosphors with a 365 nm excitation. It is noteworthy that the shoulder peaks at 596 nm, 614 nm and 660 nm arose increasingly along with the increased Eu concentration. These shoulder peaks is reasonable to attribute to the unreduced Eu^3+^ ions and can be assigned to 4f-4f transitions of Eu^3+^ (^5^D_0_-^7^F_J_ (J = 1, 2 and 3))^21^. As the doping concentration of Eu increased to 6 mol%, the emission intensity reached the maximum and then declined dramatically with a further increase of concentration. Generally, the declined intensity with increased concentration is caused by the concentration quenching effect^22^. Such effect is mainly caused by the energy consumption via energy transfer from one activator to another^23^. When the concentration of Eu increased gradually, the interatomic distance between the two Eu ions reduced, and the energy transfer rate between Eu^2+^-Eu^2+^ as well as the probability of energy transfer to luminescent killer sites increased^24^. Simultaneously, the interaction was more intensive with the reduction of interatomic distance. As a result, the *5d* band of Eu^2+^ ion decreased and led to the redshift of emission peak^25^. As depicted in Fig. 2b, an obvious redshift of emission peak occurred, indicating the intensive interaction between the identical activators^26^. However, as shown in Fig. 3, the decay curves of LaSiO_2_N:*x*Eu (*x* = 0.01–0.08) monitored at 565 nm obviously consist of two lifetimes and all decay curves could be well fitted via the second-order exponential equation^27^:


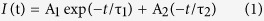


where *I* means the luminescence intensity; A_1_ and A_2_ are constants; *t* is time; and *τ*_*1*_ and *τ*_*2*_ are the lifetimes for the exponential components. As previous researches report that there is only one single La site can be substituted by Eu ion^14^,^16^. Thus, the existence of two lifetimes may due to the two kinds of decay forms for Eu:^2+^ one is the process of electrons from the excited stated to ground state; the other is the energy transfer process between Eu^2+^ and Eu^3+^ because of the coexisting of Eu^2+^ and Eu^3+^. The average lifetime τ* could be reckoned according to the following equation:


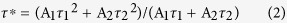


based on eqs 1 and 2, the τ* can be estimated to 2.92, 2.27, 1.87, 1.58, and 1.17 μs for LaSiO_2_N:*x*Eu with *x* = 0.01, 0.02, 0.04, 0.06 and 0.08, respectively. The donor decay times decreased as Eu concentrations increased, indicating the existence of energy transfer processes^28^,^29^. Therefore, the phenomenon of declined intensity in LaSiO_2_N:Eu might both result from the energy transfer between Eu^2+^-Eu^2+^ and Eu^2+^-Eu^3+^.

**Figure 4:**
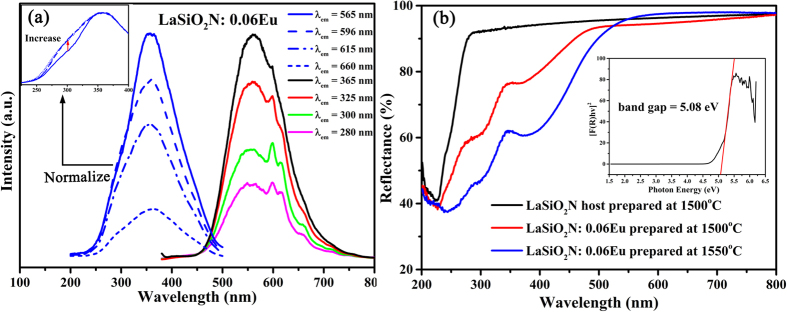
(**a**) PLE and PL spectra of the LaSiO_2_N:0.06Eu phosphor monitored by different wavelengths of emission and excitation, respectively; (**b**) Diffuse reflection spectra of LaSiO_2_N host and LaSiO_2_N:0.06Eu (prepared at 1500 °C and 1550 °C). The inset shows the absorption spectrum of LaSiO_2_N host calculated using the Kubelka-Munk equation.

The excitation spectra of LaSiO_2_N:0.06Eu were shown in Fig. 4a. Although the excitation band of Eu^3+^ were covered by that of Eu^2+^, the charge-transfer state (CTS) could be identified from the excitation spectra of LaSiO_2_N:0.06Eu under different emission features at 565 nm, 596 nm, 615 nm and 660 nm. The relative intensity of shoulder peak at around 300 nm increased from 565 nm to 660 nm excitation, suggesting it played a key role in generation of the Eu^3+^ emission. Theoretically, charge-transfer state (CTS) or the energy transfer from host lattice (HL) to Eu^3+^ can generate this shoulder peak^25^. For detailed investigation of the source of the shoulder peak, the diffuse reflectance spectra (DRS) of LaSiO_2_N host and LaSiO_2_N:0.06Eu were presented in Fig. 4b. The LaSiO_2_N host showed an energy absorption in the short-wavelength UV region (peaked at 225 nm) and a high reflection ranging from 275 to 800 nm. The band gap was estimated to be about 5.08 eV (244 nm) based on the Kubelka-Munk function^30^. When Eu ions were introduced into the LaSiO_2_N host, two broad absorption bands were observed in the 275–350 nm and 350–500 nm, demonstrating that the shoulder peak unlikely originated from HL. The emission spectra of LaSiO_2_N:0.06Eu under different excitations were also illustrated in Fig. 4a. Each emission spectrum was consist of luminescence characteristics of both Eu^2+^ and Eu^3+^ ions, and the relative intensity of Eu^3+^ reached the maximum at 300 nm, further proving the shoulder peak at 300 nm is assigned from CTS. It suggests that the coexistence of Eu^3+^ and Eu^2+^ can be realized to enhance the red region, while the emission efficiency of Eu^3+^ is insufficient to generate a marked effect.

**Figure 5:**
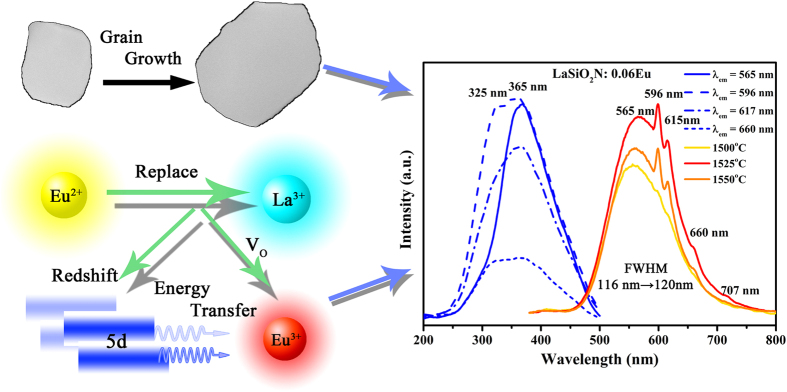
The schematic illustration of the mechanism of red enhanced Yellow-emitting LaSiO_2_N:Eu phosphor. PLE (λ_em_ = 565 nm, 596 nm, 617 nm, 660 nm ) spectra of the LaSiO_2_N:0.06Eu phosphors (prepared at 1550 °C), and PL (λ_ex_ = 365 nm) spectra of the LaSiO_2_N:0.06Eu phosphors as a function of the preparation temperature (1500 °C, 1525 °C, and 1550 °C).

**Figure 6:**
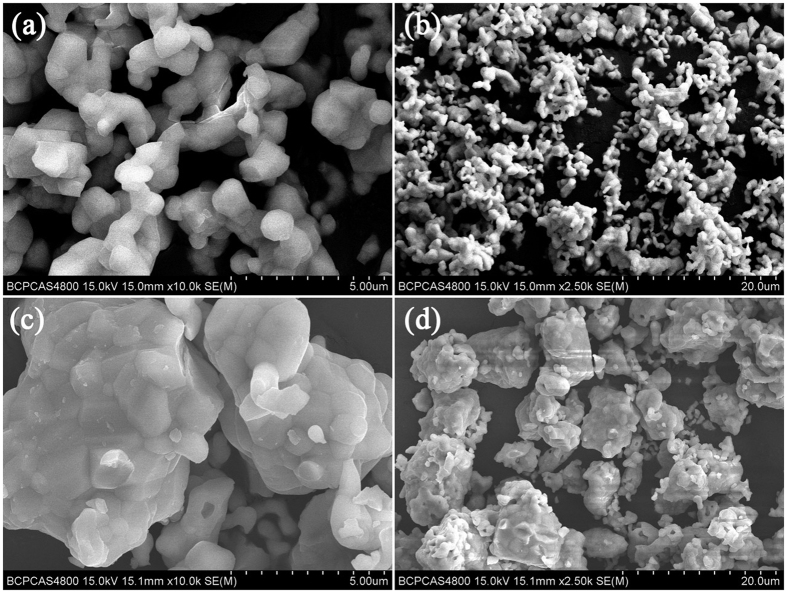
SEM images of the LaSiO_2_N:0.06Eu phosphors prepared at (**a,b**) 1500 °C and (**c,d**) 1550 °C.

For further improving the red region of LaSiO_2_N:Eu phosphor, the preparation temperature was changed, and all temperatures were controlled in the range from 1500 °C to 1550 °C to insure the obtained samples are single phase. Figure 5 shows the room temperature PLE (λ_em_ = 565 nm, 596 nm, 617 nm, and 660 nm) of the LaSiO_2_N:0.06Eu phosphors prepared at 1550 °C, and PL (λ_ex_ = 365 nm) spectra of the LaSiO_2_N:0.06Eu phosphors as a function of the preparation temperature (1500 °C, 1525 °C, and 1550 °C). The enlargement of shoulder peak (325 nm) from Eu^2+^ emission feature excitation to that of Eu^3+^ also proved the shoulder peak derived from CTS. It is interesting that not only all the emission intensities were enhanced through the increase of the preparation temperature, but also the relative intensity of Eu^3+^ characteristic peak was enhanced compared with that of Eu^2+^. One of reason for the increase of holistic emission intensity may be due to grain growth and the increased degree of crystallization at a higher preparation temperature^31^,^32^. It can be substantiated by the micro-morphology of the crystalline LaSiO_2_N:0.06Eu phosphors which were observed via SEM, TEM and XRD Refinement. As depicted in Fig. 6, the particles prepared at 1500 °C (Fig. 6a,b) had irregular morphology with the diameters ranging from 0.3 to 0.8 μm. When the preparation temperature increased, the edges and corners of irregular particles became clear, and a dramatic increase in particle sizes was observed (Fig. 6c,d). In addition, a mass of primary crystals reunited to be particles, and the obvious “sintering necks” between primary crystals suggested that grain growth occurred during the process of synthesis. The typical TEM images were illustrated in Figure S1 to further prove the grain growth. After Jade software refined, the relative crystallinity of LaSiO_2_N:0.06Eu prepared at 1500 °C and 1550 °C were estimated about 82.38% and 89.91%, respectively, demonstrating the increased degree of crystallization at a higher preparation temperature.

**Figure 7:**
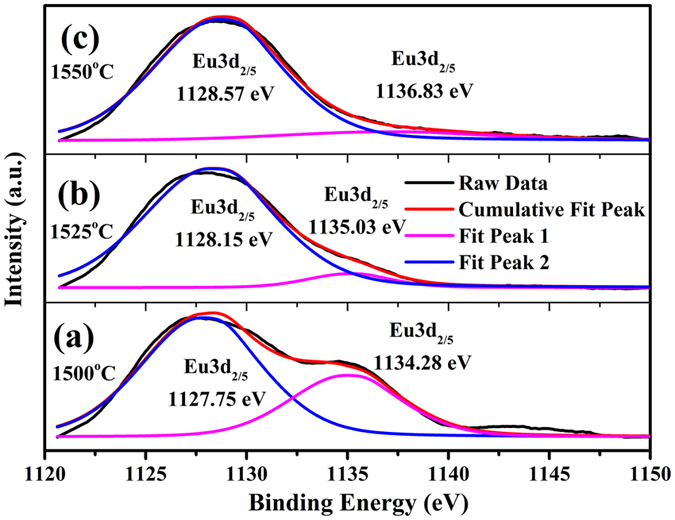
High-resolution XPS spectra at Eu 3d position of LaSiO_2_N:0.06Eu phosphors prepared at (**a**) 1500 °C, (**b**) 1525 °C and (**c**) 1550 °C.

To compare the ratio changing of Eu^2+^ and Eu^3+^, the high-resolution XPS spectra at the Eu 3d of LaSiO_2_N:0.06Eu phosphors prepared at 1500 °C, 1525 °C, and 1550 °C were detected, respectively. As exhibited in Fig. 7, two peaks were found at around 1128 eV and 1135 eV, and the shapes as well as binding energies of the Eu3d signals in LaSiO_2_N:0.06Eu agreed well with the signals of Eu^2+^ 3d_5/2_ and Eu^3+^ 3d_5/2_, respectively, indicating the existence of Eu^2+^ and Eu^3+^ ions^33^. Additionally, the relative intensity of Eu^3+^ 3d_5/2_ signals was gradually decreased with the increased preparation temperature, revealing the promotion of reduction process of Eu^3+^. This may due to the amount of thermal defects increased with the increasing preparation temperatures^34^,^35^, which could charge compensate the Eu^2+^ in the La^3+^ site and improved the reduction from Eu^3+^ to Eu^2+^ 17. Thus, the increase of Eu^2+^ concentration may be a reason for the enhanced emission intensity of Eu^2+^. However, decreased ratio of Eu^3+^ concentration compared with Eu^2+^ was inconsistent with the enhanced emission intensity of Eu^3+^. Hence, other assistance might be involved to contribute the characteristic emissions of Eu^3+^ in LaSiO_2_N.

**Figure 8:**
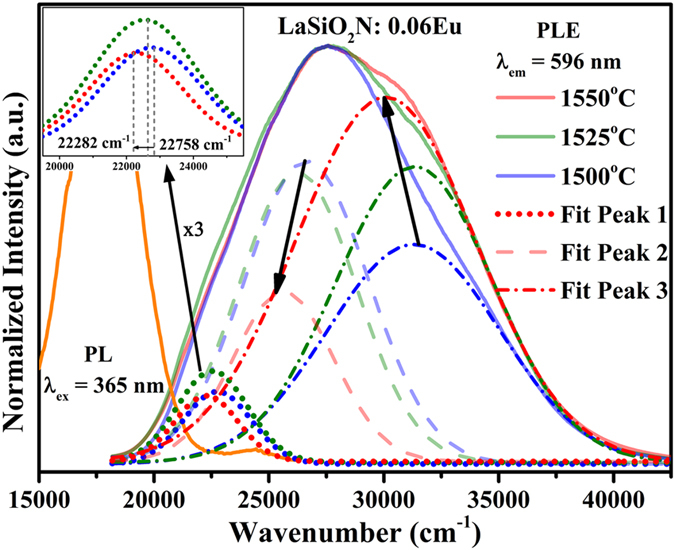
Normalized PLE spectra (λ_em_ = 596 nm) for LaSiO_2_N:0.06Eu samples (1500 °C, 1525°C, and 1550 °C) and normalized PL spectra (λ_ex_ = 365 nm) for LaSiO_2_N:0.06Eu sample. The inset shows the magnified Fit Peaks 1 of LaSiO_2_N:0.06Eu samples.

As illustrated in Fig. 8, the PLE (λ_em_ = 596 nm) spectra of the LaSiO_2_N:0.06Eu phosphors prepared at 1500 °C, 1525 °C, and 1550 °C were deconvoluted into three Gaussian components, respectively. The relative intensity of CTS (fit peak 3) was enhanced, indicating that the charge transfer from the O^2−^ to Eu^3+^ was enhanced and more efficient with the increasing preparation temperatures. This phenomenon can be explained based on the increase of oxygen vacancies (V_o_) with the increase of nonequivalent substitution of La^3+^ by Eu^2+^ in the host^25^. These V_o_ might act as sensitizers for the energy transfer from host to Eu^3+^ ion due to the strong mixing of charge transfer states^36^,^37^,^38^,^39^. Thus, the relative emission of Eu^3+^ was enhanced, and the red region of this yellow emitting phosphor was promoted. Meanwhile, an obviously redshift of CTS occurred as the preparation temperature increased, which can be attributed to the increase of Eu^3+^-O^2−^ bond length. Since the Eu^2+^ ions have smaller electronegativity than that of Eu^3+^ ions, the Eu–O bond strength became weaker with increasing concentration of Eu^2+^, resulting in weakening of bond strength. Hence the Eu^3+^-O^2-^ bond length became longer. As reported by Lin *et al.*^40^, the longer the Eu-O bond, the shorter the energy difference between the 4f and O 2p electrons, and the lower energy position of the CTB.

**Figure 9:**
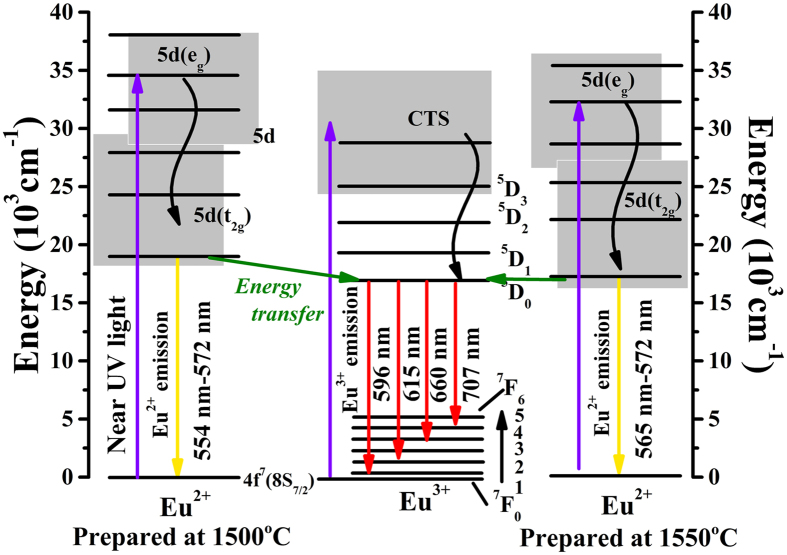
Schematic of energy transfer in LaSiO_2_N:0.06Eu.

**Figure 10:**
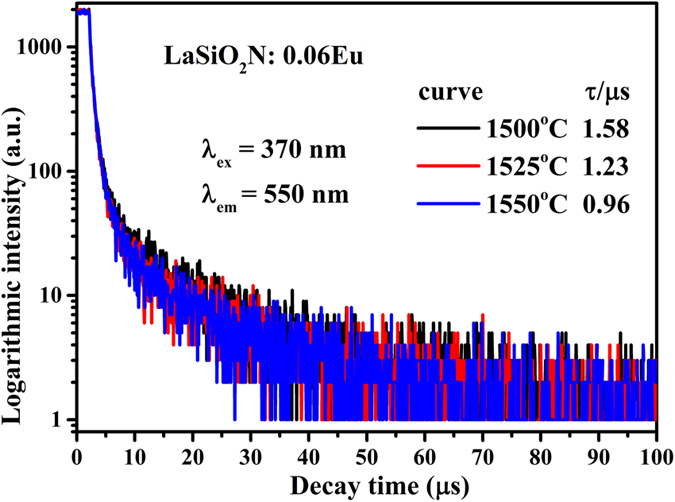
The decay curves of LaSiO_2_N:0.06Eu prepared at 1500 °C, 1525 °C, and 1550 °C, respectively.

Additionally, the coordination number of Eu^2+^ was reduced because the V_o_ increased with an increase of the Eu^2+^ content in the host, which caused the formation of a centroid of the 5d state at a lower level. Thus, as shown in Fig. 8, the 5d excitation band (fit peak 2 and 3) red shifted and the overlapping between PLE of 596 nm and PL enlarged, indicating that the energy transfer ratio between Eu^2+^ and the ^5^D_0_ level of Eu^3+^ was enhanced. The energy transfer mode between Eu^2+^ and Eu^3+^ in LaSiO_2_N: Eu was proposed in Fig. 9. Under the NUV light excitation, Eu^2+^ was excited from the ground state 4f ^7^(^8^S7/2) to the excited state 4f ^6^5d^1^. Partial energy relaxed to the ground state through the inherent transition of Eu^2+^, generating a yellow light emission; the rest of energy transferred to the nearest level ^5^D_0_ of Eu^3+^, and then 597 nm, 617 nm, 660 nm and 707 nm emissions appeared by a transition to the ^7^F_j_ (J = 1, 2, 3 and 4) ground state. With the increase of the preparation temperature, the depressed 5d level showed more overlapping with the ^5^D_0_ of Eu^3+^, resulting in the enhancement of the energy transfer between the Eu^2+^ and Eu^3+^ 25. The increased emission peak position from 550 nm at 1500 °C to 565 nm at 1550 °C and FWHM of emission peak from 116 nm at 1500 °C to 120 nm at 1550 °C also verified the redshift of 5d level^25^,^26^. The decay curves of LaSiO_2_N:0.06Eu prepared at different temperatures monitored at 565 nm were depicted in Fig. 10. All decay curves also could be well fitted via the second-order exponential equation and the lifetimes were estimated 1.58, 1.23, and 0.96 μs for LaSiO_2_N:0.06Eu prepared at 1500 °C, 1525 °C, and 1550 °C, respectively, demonstrating the existence of energy transfer. Consequently, via the increase of preparation temperature, the holistic emission intensity was enhanced owe to the increased crystallinity and reduction process. The relative emission intensity of Eu^3+^ was also increased due to the enhancement of the energy transfer. As a result, the red region of yellow emitting LaSiO_2_N: Eu phosphor was successfully enhanced.

The quantum efficiency of phosphors, which is an important technological parameter for practical application, were also been compared. The internal quantum efficiencies (IQE) of LaSiO_2_N:0.06Eu prepared at different temperature were measured and calculated by the following equations^41^:


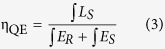


where L_S_ represents the luminescence emission spectrum of the sample; E_R_ is the spectrum of the excitation light from the empty integrated sphere (without the sample); E_S_ means the excitation spectrum for exciting the sample. As given in Figure S2, the IQE of the LaSiO_2_N:0.06Eu prepared at 1500 °C, 1525 °C and 1550 °C were estimated to be about 3.48%, 12.48% and 20.01%, respectively, under 365 nm excitation. The enhanced IQE matched well with the variation trend of emission intensity. Although the IQE of LaSiO_2_N:0.06Eu is lower than that of commercial YAG (61%)^42^, it can be further improved by optimization of the preparation conditions, because the QE depends closely on the prepared conditions, crystalline defects, particle size and morphology of the phosphor^43^,^44^.

**Figure 11:**
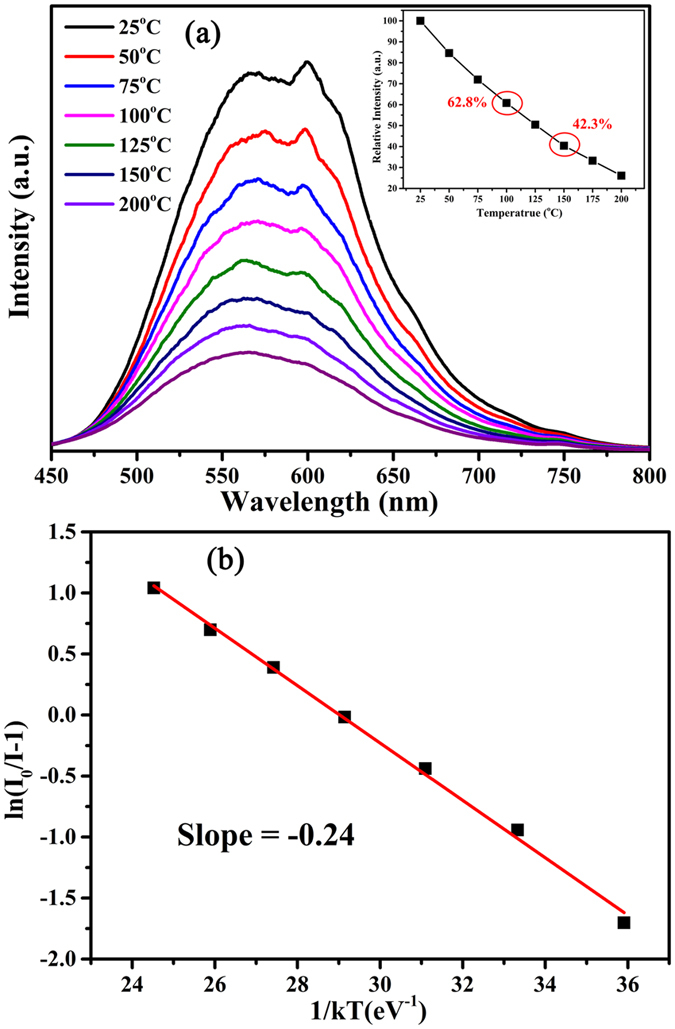
(**a**) Temperature-dependent PL spectra (λ_em_ = 365 nm) and (**b**) the plot of ln (I0/I) vs. 1/T of LaSiO_2_N:0.06Eu phosphors. The inset shows the emission intensity versus temperature.

In order to well understand the luminescent performance of this phosphor, the temperature-dependent luminescent properties of LaSiO_2_N: 0.06Eu phosphor was measured during the temperature ranges of 25–200 °C. As presented in Fig. 11a, the emission intensity decreased with increasing temperature. At 100 °C and 150 °C, the PL intensity quenched to 62.8% and 42.3%. The thermal quenching effect of this phosphor is more intense than the commercial YAG:Ce^3+^ 45, while similar to La–Si–O–N system phosphors^12^. The thermal quenching can be explained by the model of thermal excitation of the 5*d* electron to conduction band states^46^,^47^. When Eu^2+^ substituted in a trivalent site, the Eu^2+^ had the trend to be ionized to Eu^3+^, which reduced the activation energy and lead to stronger thermal quenching. To determine the activation energy for thermal quenching, the Arrhenius equation was used to estimate the thermal quenching^48^:


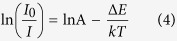


where *I*_*0*_ and *I* mean the luminescence intensity of LaSiO_2_N:Eu at room temperature and a given temperature, respectively; A is a constant; k presents the Boltzmann constant (8.617 × 10^−5^ eV K^−1^). Figure 11b plots ln[(I0/I)-1] variation dependence of 1/(kT), and the Δ*E* was calculated to be about 0.24 eV.

**Figure 12:**
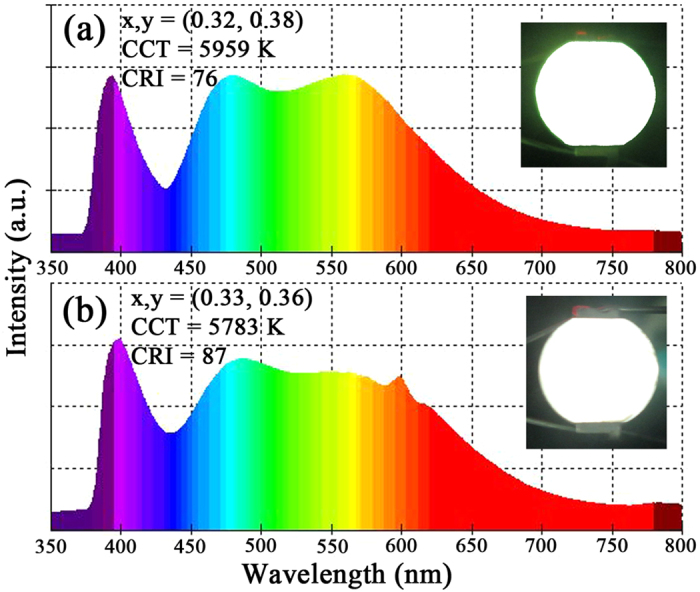
Electroluminescence spectra of W-LED lamps fabricated using a 385 nm NUV chip in combination with the mixed phosphors consisting of blue-emitting BAM:Eu^2+^ phosphor and yellow-emitting (**a**) LaSiO_2_N:0.01Eu phosphor prepared at 1500 °C, (**b**) LaSiO_2_N:0.06Eu phosphor prepared at 1550 °C. Insets show the digital images of the LED package with an input of 30 mA current.

To further assess the potential application of the LaSiO_2_N: Eu phosphors, the yellow emitting LaSiO_2_N:0.01Eu phosphor prepared at 1500 °C and LaSiO_2_N:0.06Eu phosphors prepared at 1550 °C were mixed with blue-emitting BAM: Eu^2+^ phosphor, respectively, and then the mixtures were severally combined with a 385 nm NUV chip to fabricate white LED lamps. The electroluminescent (EL) spectra of these lamps driven by 30 mA current were depicted in Fig. 12. The CIE color coordinates, CCT and CRI of the fabricated W-LED lamp with LaSiO_2_N:0.01Eu phosphor prepared at 1500 °C were determined to be (0.32, 0.38), 5959 and 76, respectively (Figure S3 and Fig. 12a). Via utilizing the redshift, varied valence and efficient energy transfer, the fabricated W-LED lamp with the red region enhanced yellow-emitting LaSiO_2_N:0.06Eu phosphor displayed an entire white spectrum with a CIE color coordinates of (0.33, 0.36), a CCT of 5783 K and a CRI of 87 (Figure S3 and Fig. 12b). Compared with the W-LED lamp using commercial YAG:Ce phosphor in previous study (CIE = 0.302, 0.315; CCT = 7272 K, Ra = 78.38)^3^, the CCT of as-fabricated LEDs was relative low and the Ra was high, suggesting that the high CRI warm W-LEDs could be easily obtained by altering the concentration of doping ions and the preparation temperature.

## Conclusions

In summary, a novel Eu-activated LaSiO_2_N yellow-emitting phosphor has been synthesized and evaluated for the application in W-LEDs. With the aid of crystal structure, valence-varied, redshift, and energy transfer, the red region enhanced yellow-emitting LaSiO_2_N: Eu phosphor has been designed and realized by controlling the concentration of doping ions and the preparation temperature. A high CRI and warm W-LED lamp was obtained in combination with this phosphor, proving that the red region enhanced LaSiO_2_N: Eu phosphor has a great potential for the application in W-LEDs. More importantly, this study would provide a new strategy for designing Eu-activated yellow-emitting phosphors by synergistically enhancing the red region and emission intensity to adjust the CCT and CRI for warm W-LEDs application without reducing the other luminescence properties.

## Methods

### Materials and Synthesis

The LaSiO_2_N:Eu was synthesized from stoichiometric mixtures of La_2_O_3_ (analytical reagent (A. R.)), α-Si3N4 (A. R.) and Eu_2_O_3_ (A. R.). The ground powders were placed in alumina crucibles and fired for 6 h in a reducing atmosphere (10% H_2_ + 90% N_2_) at 1500 °C, 1525 °C and 1550 °C, respectively. Then, the precursor was reground and heated again at same condition. Thereafter, the samples were cooled down to room temperature naturally and powdered for subsequent analysis.

### Materials Characterization

Powder X-ray diffraction on a D8 Advance diffractometer (Germany) with graphite-monochromatized Cu Kα radiation (λ = 0.154 06 nm) was recorded for the structure of all samples. Photoluminescence spectra were collected using a Hitachi F-4600 fluorescence spectrophotometer (Japan) equipped with a 150 W Xe lamp as the excitation source. Diffuse reflection spectra were recorded using a Shimadzu UV-3600 UV−vis−NIR spectrophotometer attached with an integrating sphere. BaSO4 was used as a reference for 100% reflectance. The morphology was observed using scanning electron microscopy (SEM; JSM-6460LV, JEOL, Japan). X-ray photoelectron spectroscopy (XPS) measurements were performed in a PHI 5300 ESCA system using an Al Ka X-ray source with constant pass energy of 55.00 eV. The charge effect referred to the C1s signal (284.6 eV). The room-temperature luminescence decay curves were obtained from a spectrofluorometer (Horiba, Jobin Yvon TBXPS) using a tunable pulse laser radiation (nano-LED) as the excitation. Quantum efficiency was measured by a fluoromax-4 spectrofluorometer (Horiba, Jobin Yvon) with an integral sphere at room temperature.

## Additional Information

**How to cite this article**: Chen, J. *et al.* Design of a Yellow-Emitting Phosphor with Enhanced Red Emission via Valence State-control for Warm White LEDs Application. *Sci. Rep.* **6**, 31199; doi: 10.1038/srep31199 (2016).

## Supplementary Material

Supplementary Information
