# Supplementary material for: Design of a Yellow-Emitting Phosphor with Enhanced Red Emission via Valence State-control for Warm White LEDs Application
Source: Sci Rep. 2016 Aug 11;6:31199. doi: 10.1038/srep31199 (PMC4980616; doi:10.1038/srep31199)
Supplement: Supplementary Information [file srep31199-s1.doc]

**Supporting information**

**Design of a Yellow-Emitting Phosphor with Enhanced Red Emission via Valence State-control for Warm White LEDs Application**

**Jian Chen,^1^** **Yangai Liu*,^1^ Lefu Mei,^1^ Peng Peng,^2^ Qijin Cheng^3^ & Haikun Liu^1^**

^1^*Beijing Key Laboratory of Materials Utilization of Nonmetallic Minerals and Solid Wastes, National Laboratory of Mineral Materials, School of Materials Science and Technology, China University of Geosciences, Beijing 100083, china,*^2^*Department of Materials Processing and Control Engineering, School of Mechanical Engineering and Automation, Beihang University, Beijing, 100191, China,*^3^*School of Energy Research, Xiamen University, Xiamen 361005, Fujian, China.*

Correspondence and requests for materials should be addressed to liuyang@cugb.edu.cn.

**
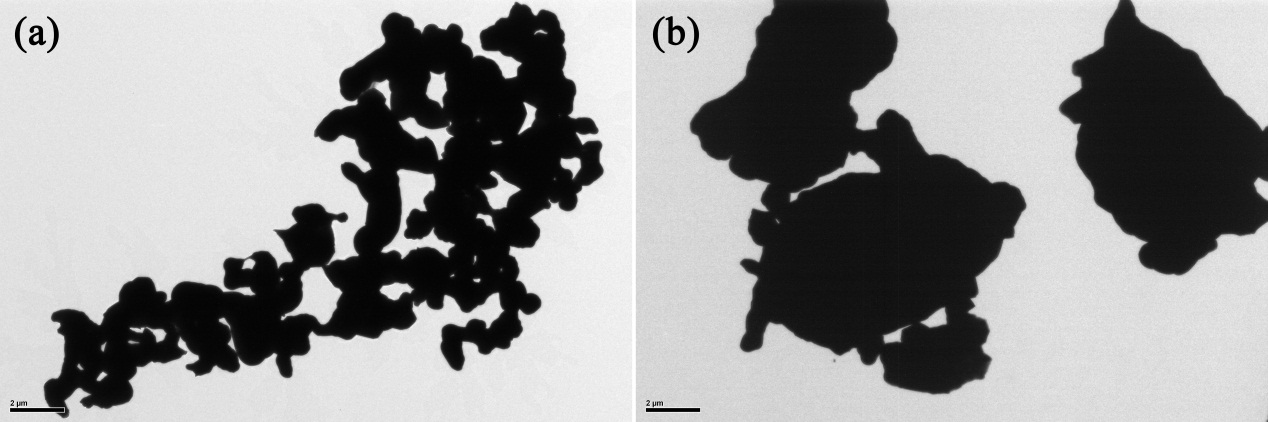
**

**Figure S1 |** TEM images of the LaSiO_2_N:0.06Eu phosphors prepared at (a) 1500^o^C and (b) 1550^o^C.


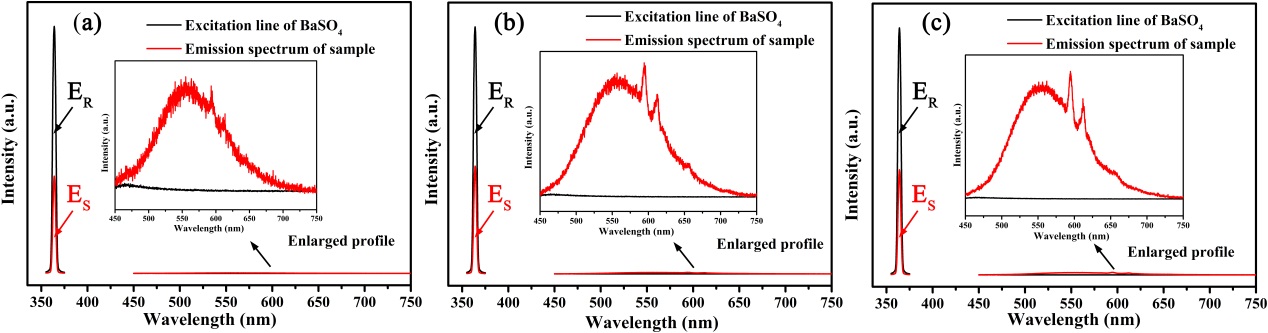


**Figure S2 |** Excitation line of BaSO_4_ and emission spectrum of the LaSiO_2_N:0.06Eu phosphor prepared at (a) 1500^o^C, (b)1525^o^C and (c)1550^o^C collected by an integrating sphere. The inset shows the magnification of the emission spectrum.


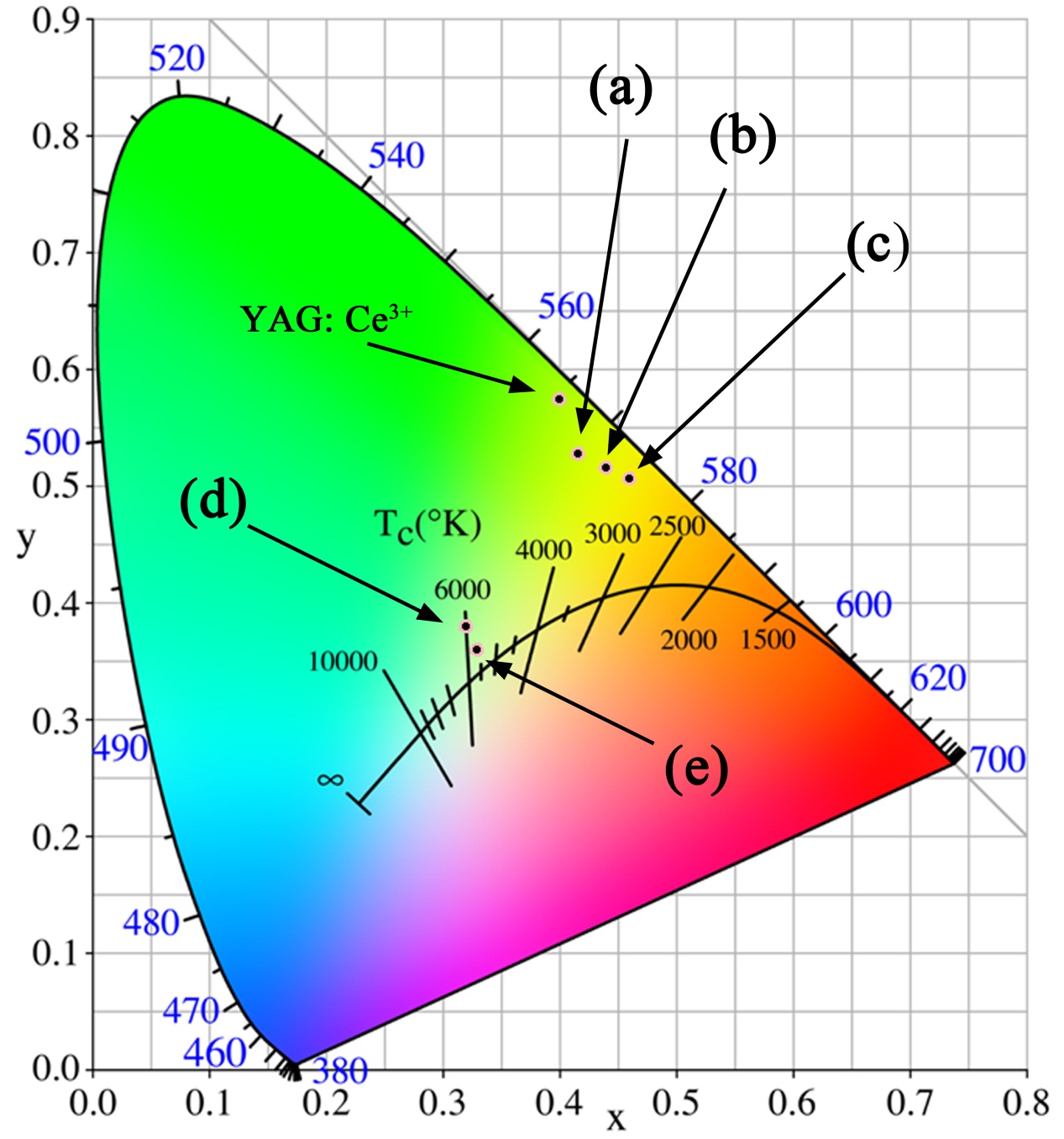


**Figure S3 |** Chromaticity coordinate of (a) LaSiO_2_N:0.01Eu phosphor prepared at 1500^o^C, (b) LaSiO_2_N:0.06Eu phosphor prepared at 1525^o^C, (c) LaSiO_2_N:0.06Eu phosphor prepared at 1550^o^C and WLEDs fabricated by combining blue-LED chip with (d) LaSiO_2_N:0.01Eu phosphor prepared at 1500^o^C, (e) LaSiO_2_N:0.06Eu phosphor prepared at 1550^o^C in the Commission Internationale de IʹÉclairage (CIE) 1931 color spaces.
